# Digital Divide in Perceived Benefits of Online Health Care and Social Welfare Services: National Cross-Sectional Survey Study

**DOI:** 10.2196/17616

**Published:** 2020-07-07

**Authors:** Tarja Heponiemi, Vesa Jormanainen, Lars Leemann, Kristiina Manderbacka, Anna-Mari Aalto, Hannele Hyppönen

**Affiliations:** 1 Finnish Institution for Health and Welfare Helsinki Finland

**Keywords:** digital divide, online services, benefits, skills, access, sociodemographics, participation, Finland

## Abstract

**Background:**

The number of online services in health care is increasing rapidly in developed countries. Users are expected to take a more skilled and active role in taking care of their health and prevention of ill health. This induces risks that users (especially those who need the services the most) will drop out of digital services, resulting in a digital divide or exclusion. To ensure wide and equal use of online services, all users must experience them as beneficial.

**Objective:**

This study aimed to examine associations of (1) demographics (age, gender, and degree of urbanization), (2) self-rated health, (3) socioeconomic position (education, experienced financial hardship, labor market position, and living alone), (4) social participation (voting, satisfaction with relationships, and keeping in touch with friends and family members), and (5) access, skills, and extent of use of information and communication technologies (ICT) with perceived benefits of online health care and social welfare services. Associations were examined separately for perceived health, economic, and collaboration benefits.

**Methods:**

We used a large random sample representative of the Finnish population including 4495 (56.77% women) respondents aged between 20 and 97 years. Analyses of covariance were used to examine the associations of independent variables with perceived benefits.

**Results:**

Access to online services, ICT skills, and extent of use were associated with all examined benefits of online services. ICT skills seemed to be the most important factor. Poor self-rated health was also consistently associated with lower levels of perceived benefits. Similarly, those who were keeping in touch with their friends and relatives at least once a week perceived online services more often beneficial in all the examined dimensions. Those who had experienced financial hardship perceived fewer health and economic benefits than others. Those who were satisfied with their relationships reported higher levels of health and collaboration benefits compared with their counterparts. Also age, education, and degree of urbanization had some statistically significant associations with benefits but they seemed to be at least partly explained by differences in access, skills, and extent of use of online services.

**Conclusions:**

According to our results, providing health care services online has the potential to reinforce existing social and health inequalities. Our findings suggest that access to online services, skills to use them, and extent of use play crucial roles in perceiving them as beneficial. Moreover, there is a risk of digital exclusion among those who are socioeconomically disadvantaged, in poor health, or socially isolated. In times when health and social services are increasingly offered online, this digital divide may predispose people with high needs for services to exclusion from them.

## Introduction

### Background

The number of online services in health care is increasing rapidly in Finland and other developed countries. The new online services are implemented and adopted to support self-management and self-service, and thus users are expected to take a more skilled and active role in maintaining their well-being, health, and especially long-term illnesses. To ensure that users increase participation in online services, it is important that they find that online services are beneficial for them [1].

There are risks that users (especially those people who need the services most) will drop out of online services resulting in a digital divide or exclusion. The concept of digital divide has traditionally referred to the gap between persons who have and do not have access to new forms of information technology [2]. Limits to access can have motivational, physical, skills, or use grounds [3]. Nonuse of online digital services can have psychological (motivation, confidence, and experienced attractiveness of use), physical (opportunity to use), or practical (lack of skills) causes [4]. As the internet has diversified and diffused widely over the past 10 years, it is assumed that internet nonuse is, at present, far more common among vulnerable groups and that lack of access has become a less important reason for internet nonuse compared with lack of skills [5].

The digital divide or exclusion affects the poorest and most marginalized groups from the outset [6]. The internet reinforces existing social differences, and internet nonusers are reported to be older and less educated and more likely to be unemployed, disabled, and socially isolated [7]. Sociodemographic, economic, and geographical factors have been found to be key determinants of digital exclusion [8]. Among urban adults, the discriminating factors between computer users and nonusers include age, education, employment, and income, self-rated health status, hospital visits, organization membership, and participation in voluntary work [9].

A previous study from the Netherlands showed that the elderly benefit less from internet use than the young [5]. Moreover, those less educated and poorer gained fewer economic benefits from the internet than their counterparts, and single people gained fewer social benefits than those living with others [5]. A study from the United Kingdom found that more educated people benefit most from internet use [10]; surprisingly, the elderly benefited more than the young. In Germany, it was found that enabling and caring support through the internet was associated with job search benefits among the unemployed [11].

Social and digital exclusion are interlinked: differential access to technology contributes to socioeconomic stratification or inclusion and vice versa [12]. The debate has moved from a single digital divide to multiple digital divides that include not only global challenges but also local contextual problems in terms of availability of content, bandwidth, and skills, among other issues [13]. Helsper’s [14] corresponding fields model suggests that digital and social exclusion influence one another and the resources a person has offline influence their ability to use digital online solutions. Consequently, client and patient resources influence adoption and sustained use of new digital online health services. Therefore, socioeconomic factors, social participation, health status, and well-being have potential effects on digital divide or exclusion. Only by studying these together can we identify the separate and combined inﬂuences of different types of social exclusion on different types of digital exclusion. The model characterizes access and skills as important social impact mediators in this context. Without access, the internet or other information and communication technologies (ICT) cannot be used, which makes access the most basic mediator between offline and digital fields of exclusion. Similarly, certain skills on a basic technical and operational level are required for the use of ICT and the internet [14].

Aceto et al [15] have suggested that ICT in health care decreases costs, enables discovery, and improves patient outcomes. Moreover, ICT is proposed to extensively transform health care to being more proactive, preventive, and person-centered instead of being reactive and hospital-centered [15]. To fully take advantage of all the suggested benefits of ICT, patients must use online services and find them beneficial. We need to understand why people choose not to take advantage of the opportunities ICT offers for managing their health and define the characteristics of people who are most likely to benefit and those at risk of exclusion.

Research focus should shift from access to digital technology (first-level divide) to skills and use of technology (second-level divide) and who benefits the most or least from internet use (third-level divide) [16,17]. Third-level digital divide refers to gaps in capacity to translate individual internet access and use into favorable offline outcomes [5]. Research related to the third-level divide and its social determinants is scarce [17], and the role of access, skills, and extent of use in this context must be identified. Previous studies [4,7,8] have focused on general use of the internet, but information about determinants of using the internet for health care and social welfare services is lacking and must be identified because these vital services are increasingly moving online.

### Objectives

This study aimed to examine direct associations of demographics (age, gender, degree of urbanization), self-rated health, socioeconomic position (education, experienced financial hardship, labor market position, and living alone) and social participation (voting, satisfaction with relationships, and keeping in touch with friends and family members) with perceived benefits of online health care and social welfare services (health, economic, and collaboration benefits). The study data are based on a large random sample representative of the Finnish population. Furthermore, we examined the effects of access, skills, and the extent of ICT use and whether these could account for those associations.

## Methods

### Sampling

The data collection and questionnaire formulation have been reported in more detail elsewhere [18]. A random sample of 10,000 people representative of the adult population (aged 20 years and older) living in Finland was collected from the Population Register Center of Finland. For those aged 75 years and older, double picking probability was used in order to guarantee sufficient group size. The questionnaire (Multimedia Appendix 1) was sent by postal mail to all sampled persons in 2017 with instructions on how to respond online. Reminders were sent 3 times to those who had not responded. The response rate was 44.95% (4495/10,000), with 56.77% (2552/4495) being women. The respondents were older, more often women, and had more education than the eligible population [18], and we addressed this by applying inverse probability weighting based on age, gender, marital status, education level, living region, and degree of urbanization of the residential municipality. This method has been used previously in many Finnish population studies, and it has been found to perform well in correcting possible effects of nonresponse on representativeness of the results [19].

### Setting

Finland is among the leading countries in digitalization of health care and social welfare services. The country has launched nationwide integrated data services for health care and social welfare services, the Kanta services, in several steps from May 2010 onward [20,21]. Kanta services are targeted to citizens, health care and social welfare service providers and professionals, and community pharmacies. Kanta services include electronic prescribing (Prescription Centre), patient-accessible electronic health records (My Kanta Pages), health records (Patient Data Repository), and the client data archive for social welfare services. It is mandatory for all health care providers, public or private, to subscribe to and use the national Kanta services. Implementation and adoption of Kanta services for social welfare started in May 2018 and is currently ongoing. In addition to national comprehensive undertakings, at least two other nationwide digital service projects (Health Village 2.0 and Digisote [former ODA]) have been developed, implementing a large number of new online health and welfare services. In particular, these services have focused on supporting clients to self-manage their health and well-being online. Moreover, service providers such as hospital districts and primary health care centers provide regional and local electronic online services such as booking services, electronic consultations, and web-based question-answer services, etc [22].

### Measurements

#### Dependent Variables

Benefits of online health and social care services were measured with 3 scales on perceived health, economic, and collaboration benefits. Health benefits were measured with 6 items such as “online services help citizens self-manage their health/well-being.” Cronbach alpha for this scale was α=.90. Economic benefits included 4 items (α=.87) such as “online services provide useful reminders (eg, the time of reception, laboratory tests, renewing prescriptions etc).” Collaboration benefits included 3 items (α=.86) such as “online services support the collaboration and information flow between the patient/client and carer.” All items were rated on a 5-point Likert scale ranging from 1 (completely disagree) to 5 (completely agree), and the mean for each scale was calculated.

#### Independent Variables

Demographics included age in years, gender (male or female), and degree of urbanization of the residential municipality (urban, semiurban, or rural area). Health status was measured by asking how respondents would describe their state of health at present (with answer alternatives good, fairly good, average, fairly poor, poor). For analyses, it was coded 0 (above average health) or 1 (average health or poorer).

Indicators of socioeconomic position were education, classified into 3 categories (low, intermediate, or high) by setting the self-reported years of education in proportion to the tertiles of education years in each respondents’ age and gender group (using 10-year age groups); financial hardship, assessed by asking whether respondent had been forced to bargain for food, medication, or physician visits due to shortage of money during the previous 12 months (none or yes to at least one of these); current labor market position (currently working, old age pension, disability pension, unemployed, student, or other); and living alone (no or yes).

Indicators of social participation were voted in last municipal elections (no or yes), satisfaction with one’s social relationships (no or yes), and keeping in contact with friends and relatives outside the own household at least once a week (no or yes).

ICT-related variables included access to, skills to use, and extent of use related to online services in health and social care. Access to online services was measured by 7 items (α=.84) asking availability of online services, access to computer and internet, quality of data connections, possibility of getting online services in their own language, accessibility of services despite obstacles (eg, disabilities), ease of finding online services, and possibility of taking care of things online on behalf of someone else. Skills to use online services were measured with 5 items (α=.86) assessing technical skills to use online services, support received for using online services, possibility of receiving help when facing technical problems, experienced clearness and length of terms of use, and perceived ease of using online services. The response options for access and skills scales ranged between 1 (completely disagree) and 5 (completely agree), and the mean of the items was calculated for both scales. The extent of use of online services in health and social care was measured with 16 items asking whether the respondent had used described service/functionality during the past year (response options no, yes traditionally, or yes online). The functionalities/services were related to self-care (3 items assessing, for example, whether the respondent had used online symptom checkers or followed own personal health data online), use for collaboration (8 items such as whether respondent has viewed the patient data recorded by professionals about them, received laboratory results, renewed prescriptions, or asked advice from professionals), and seeking out services (5 items assessing, for example, whether the respondent had searched for information about available services, booked appointment, or compared quality of services). The sum of functionalities/services a respondent had used online was calculated, resulting in a range of 0 to 16 functionalities (extent of use).

### Ethical Issues

Ethical approval for the study was received from the research ethics committee of the Finnish Institute for Health and Welfare. Data were collected with no direct identification information concerning the respondents, and therefore no individuals can be identified from the data.

### Statistical Analysis

Associations of independent variables with outcome variables related to perceived benefits (health, economic, and collaboration) were examined using analysis of covariance (in separate analyses). Analyses were conducted in 3 steps. In the first step, analyses included demographics (age, gender, and degree of urbanization) and health status (model A). In the second step, variables related to socioeconomic position (education, experienced financial hardship, labor market position, living alone) and variables related to social participation (voting, satisfaction with relationships, and keeping in touch with friends and family members) were added to the model (model B). In model C, ICT-related variables concerning access, skills, and the extent of use were added. Skills and access were examined in separate analyses to avoid multicollinearity because they correlated with each other. Analyses were conducted in these steps to examine the independent effect of each of the demographic and socioeconomic variables on each of the perceived benefits and whether ICT-related variables would partly account for possible associations of the independent variables from models A and B with outcome variables. Analyses were conducted using SPSS Statistics version 25 (IBM Corp). Methods suitable for weighted data were used (ie, complex samples general linear model for analyses of variance and complex samples descriptives and frequencies for descriptive statistics).

## Results

### Characteristics of Respondents

The characteristics of the study population are presented in Table 1. The mean age of the respondents was 51.7 years, more than 70% (3201/4494, 71.23%) lived in urban regions, and about half (2160/4495, 48.08%) were employed. Those respondents having low education comprised the largest group. Approximately two-thirds (3054/4495, 67.94%) of the respondents had used at least one of the online health or social services and functionalities under study.

**Table 1:** The basic background characteristics of the weighted study sample (n=4495).

Characteristics		Value
**Gender^a^, n (%)**		
	Women	2300 (51.18)
	Men	2194 (48.82)
**Degree of urbanization, n (%), (n=4494)**		
	Urban	3201 (71.23)
	Semiurban	658 (14.64)
	Rural	635 (14.13)
**Self-rated health, n (%)**		
	Above average	2884 (64.83)
	Average or poorer	1564 (35.17)
**Education, n (%), (n=4260)**		
	Low	1888 (44.32)
	Intermediate	1279 (30.02)
	High	1093 (25.66)
**Financial hardship, n (%)**		
	No	3613 (81.7)
	Yes	807 (18.3)
**Labor market position, n (%), (n=4494)**		
	Working	2160 (55.34)
	Old age pension	823 (21.09)
	Disability pension	158 (4.05)
	Unemployed	273 (7.00)
	Student	257 (6.58)
	Other	232 (5.94)
**Living alone, n (%)**		
	No	3174 (72.19)
	Yes	1223 (27.81)
**Voted in the last municipal elections, n (%)**		
	No	1142 (26.20)
	Yes	3217 (73.80)
**Satisfaction with relationships, n (%)**		
	No	984 (22.68)
	Yes	3355 (77.32)
**Keeping in touch with friends and relatives, n (%)**		
	No	805 (18.09)
	Yes	3645 (81.91)
Age^b^, mean (SE)		51.7 (0.32)
**ICT^c^-related factors**		
	Access^d^, mean (SE)	3.90 (0.02)
	Skills^e^, mean (SE)	3.51 (0.02)
	Extent of use^d^, mean (SE)	2.62 (0.05)

^a^Because this table presents weighted results, the percentages of genders vary from those given for the actual respondents (eg, in the abstract and sampling section).

^b^Range: 20-97.

**^c^**ICT: information and communication technologies.

^d^Range: 1-5.

^e^Range: 0-16.

### Perceived Health Benefits

Table 2 shows results regarding perceived health benefits. In model A, age and self-rated health were significantly associated with perceived health benefits. Older respondents and those with poor health perceived fewer health benefits from online services. The association of health to perceived benefits remained significant after all adjustments, whereas the association regarding age attenuated to nonsignificant after adjusting for ICT-related variables. In model B, education, financial hardship, satisfaction with relationships, and keeping in touch with friends and relatives were all significantly associated with health benefits. Compared with other respondents, perceived health benefits were higher among those who were satisfied with their relationships and were keeping in touch with their friends and relatives, whereas health benefits were lower among those who had experienced financial hardship. Those having high education (estimated mean [EM] 3.64 [SE 0.04]) or intermediate education (EM 3.62 [SE 0.04]) perceived more health benefits compared with those having low education (EM 3.48 [SE 0.04]). Other associations persisted after adding the ICT-related variables, whereas education did not remain significant after that. In model C, higher levels of access, skills, and extent of use were all associated with higher levels of perceived health benefits. *R*^2^ of model C was .16.

**Table 2:** Association of explanatory factors with perceived health benefits (analysis of covariance, n=4495).

Variable	Model A		Model B		Model C	
	*F* score (*df*)	*P* value	*F* score (*df*)	*P* value	*F* score (*df*)	*P* value
Gender	0.84 (1)	.36	1.06 (1)	.30	2.61 (1)	.11
Age	37.93 (1)	<.001	11.48 (1)	.001	1.70 (1)	.19
Urbanization	1.09 (2)	.34	1.11 (2)	.33	1.79 (2)	.17
Self-rated health	53.33 (1)	<.001	16.24 (1)	<.001	1.94 (1)	.001
Education	—	—	1.70 (2)	<.001	2.74 (2)	.07
Financial hardship	—	—	5.97 (1)	.02	6.17 (1)	.01
Labor market position	—	—	1.02 (5)	.40	0.77 (5)	.57
Living alone	—	—	0.00 (1)	.97	0.37 (1)	.54
Voting	—	—	3.06 (1)	.08	8.15 (1)	.004
Relationships	—	—	8.52 (1)	.004	6.75 (1)	.009
Keeping in touch	—	—	17.91 (1)	<.001	9.27 (1)	.002
Access	—	—	—	—	51.43 (1)	<.001
Skills	—	—	—	—	109.76 (1)	<.001
Extent of use	—	—	—	—	43.02 (1)	<.001

### Perceived Economic Benefits

Table 3 shows the results regarding perceived economic benefits. In model A, age, degree of urbanization, and health status were significantly associated with perceived economic benefits. Older respondents and those with poor health perceived fewer economic benefits compared with their counterparts. Those living in urban areas perceived more economic benefits (EM 4.1 [SE 0.02]) than those living in semiurban (EM 3.9 [SE 0.04]) or rural areas (EM 4.0 [SE 0.04]). The association of benefits with health remained significant after all adjustments, whereas the association with age attenuated to nonsignificant after model B adjustments and with urbanization after adding ICT-related variables. In model B, education, financial hardship, and keeping touch with friends and relatives were all significantly associated with perceived economic benefits. Those having high (EM 3.94 [SE 0.05]) or intermediate (EM 3.96 [SE 0.04]) education perceived more economic benefits compared with those having low education (EM 3.83 [SE 0.04]). Perceived economic benefits were higher among those who were keeping in touch with their friends and relatives and lower among those who had experienced financial hardship. The associations of economic benefits with financial hardship and keeping in touch with friends and relatives remained significant in all analyses, whereas the association with education attenuated to nonsignificant after adjusting for ICT-related variables. In model C, higher levels of access, skills, and extent of use were all associated with higher levels of perceived economic benefits. *R*^2^ of model C was .14.

**Table 3:** The association of explanatory factors with perceived economic benefits (analysis of covariance, n=4495).

Variable	Model A		Model B			Model C		
	*F* score (*df*)	*P* value		*F* score (*df*)	*P* value		*F* score (*df*)	*P* value
Gender	0.90 (1)	.34		0.95 (1)	.33		0.05 (1)	.82
Age	17.37 (1)	<.001		0.54 (1)	.46		2.10 (1)	.15
Urbanization	4.13 (2)	.02		3.74 (2)	.02		2.57 (2)	.08
Self-rated health	51.42 (1)	<.001		11.65 (1)	.001		8.07 (1)	.005
Education	—	—		7.67 (2)	<.001		1.93 (2)	.15
Financial hardship	—	—		9.85 (1)	.002		9.64 (1)	.002
Labor market position	—	—		2.00 (5)	.08		0.87 (5)	.50
Living alone	—	—		1.01 (1)	.32		0.04 (1)	.84
Voting	—	—		0.07 (1)	.79		2.21 (1)	.14
Relationships	—	—		0.59 (1)	.44		0.48 (1)	.49
Keeping in touch	—	—		15.39 (1)	<.001		6.84 (1)	.009
Access	—	—		—	—		58.32 (1)	<.001
Skills	—	—		—	—		93.14 (1)	<.001
Extent of use	—	—		—	—		54.10 (1)	<.001

### Perceived Collaboration Benefits

Table 4 shows the results regarding perceived collaboration benefits. In model A, age and health status were significantly associated with collaboration benefits. Older respondents and those with poor self-rated health perceived fewer collaboration benefits. The association between health and perceived collaboration benefits remained significant after all adjustments, whereas the association with age attenuated to nonsignificant after adjusting for ICT-related variables. In model B, satisfaction with relationships and keeping in touch with friends and relatives were both significantly associated with collaboration benefits. Perceived collaboration benefits were higher among those who were satisfied with their relationships and those who were keeping in touch with their friends and relatives. These associations remained significant even after adjusting for ICT-related variables. In model C, higher levels of access, skills, and extent of use were all associated with higher levels of perceived collaboration benefits. *R*^2^ of model C was .15.

**Table 4:** The association of explanatory factors with perceived collaboration benefits (analysis of covariance, n=4495).

Variable	Model A		Model B			Model C		
	*F* score (*df*)	*P* value		*F* score (*df*)	*P* value		*F* score (*df*)	*P* value
Gender	0.20 (1)	.65		0.00 (1)	.99		0.38 (1)	.54
Age	24.46 (1)	<.001		4.55 (1)	.03		0.00 (1)	.99
Urbanization	2.63 (2)	.07		3.04 (2)	.048		3.29 (2)	.04
Self-rated health	48.81 (1)	<.001		14.48 (1)	<.001		1.26 (1)	.001
Education	—	—		2.10 (2)	.12		0.54 (2)	.58
Financial hardship	—	—		2.46 (1)	.12		2.23 (1)	.14
Labor market position	—	—		0.57 (5)	.72		0.30 (5)	.91
Living alone	—	—		1.78 (1)	.18		0.46 (1)	.50
Voting	—	—		1.73 (1)	.19		5.43 (1)	.02
Relationships	—	—		7.68 (1)	.006		6.86 (1)	.009
Keeping in touch	—	—		25.71 (1)	<.001		15.05 (1)	<.001
Access	—	—		—	—		56.14 (1)	<.001
Skills	—	—		—	—		111.77 (1)	<.001
Extent of use	—	—		—	—		38.90 (1)	<.001

### Additional Analyses

Given that ICT-related variables attenuated the association of age, education, and urbanization to nonsignificant, we additionally examined the associations between these variables and access, skills, and extent of use in separate analyses of variance. All associations were statistically highly significant. Older respondents reported lower levels of access (*F*_1_=842.8, *P*<.001), skills (*F*_1_=1069.5, *P*<.001), and extent of use (*F*_1_=266.2, *P*<.001) compared with younger respondents. Those who were living in urban areas reported higher levels of access (*F*_2_=37.2, *P*<.001), skills (*F*_2_=35.7, *P*<.001), and extent of use (*F*_2_=29.9, *P*<.001) compared with those living in semiurban or rural areas. Those with a low education level reported lower levels of access (*F*_2_=47.5, *P*<.001), skills (*F*_2_=39.4, *P*<.001), and extent of use (*F*_2_=36.1, *P*<.001) compared with those having intermediate or high education.

## Discussion

### Principal Findings

This study examined the associations of demographics, health, socioeconomic position, social participation, and ICT variables with perceived benefits of using online services. We found that access to online services, ICT skills, and extent of use were consistent factors associated with all examined benefits (ie, with perceived health, economic, and collaboration benefits). ICT skills seemed to be the most important factor. Moreover, self-rated poor health was consistently associated with lower levels of perceptions of each of the benefits examined. Experienced financial hardship seemed to be the most important indicator of socioeconomic position, given that poor people who reported having been forced to bargain for basic needs such as food, medication, or physician visits due to financial constraints perceived fewer health and economic benefits from online services. Dimensions of social participation were also highly relevant to perceived benefits. Those who were keeping in touch with their friends and relatives at least once a week perceived online services beneficial regarding all dimensions examined. Those who were satisfied with their relationships reported higher levels of health and collaboration benefits compared with other respondents.

Older respondents perceived less of each of the benefits examined, but this seemed to be at least partly explained by their lower access to online services, poorer skills when using these services, and lower extent of use. Those living in urban areas perceived more economic benefits than others, but this seemed to be at least partly explained by their higher access, skills, and use. Gender was unrelated to perceived benefits.

In addition to experienced financial hardship, there were also significant associations of other dimensions of socioeconomic position with the examined benefits. Those with a low level of education perceived lower levels of health and economic benefits. However, these significant associations also attenuated to nonsignificant after adjusting for ICT variables suggesting that the effects of education on perceived benefits from online services may also be largely explained by low access, poor skills, and low use among those with a lower level of education.

### Comparison With Previous Results

This study showed the importance of ICT access, skills, and the extent of use for perceived benefits of online services. Previous studies have mainly focused on the determinants of these variables but not on their role for the outcomes such as perceived benefits [9,23]. Access and digital skills have been identified as being vital for the success of personal health record use and for ensuring that the records will not become limited to those who are already linked to the internet with high levels of health literacy and computer skills [24]. Internet use has previously been associated with economic, social, and institutional benefits [5]. Access and skills have also been associated with the adoption, engagement, and sustained use of patient portals [25-27].

Our findings suggest that people with economic disadvantage, poor health, and a low level of participation see fewer benefits from online services even when differences in access, skills, and use are accounted for. This corresponds to a previous finding showing that those with below-average earnings gained fewer economic benefits from the internet use than those with average earnings [5]. It is possible that people from these disadvantaged groups need more personal and individually tailored health and social services, which evokes an urge for getting services provided in an interpersonal encounter. Online services may be more suited for providing care for uncomplicated problems in a standardized manner and prevention of health or welfare problems. The health and welfare problems of disadvantaged groups may tend to be more complex, including multimorbidity, and potentially include social problems. Thus, they may require multisectoral and multidisciplinary services and simultaneous, coordinated action of different professionals.

Our results suggest that older people experience fewer benefits from online services, but this association is partly due to their lower levels of access, poorer skills, and lower use of online services. Previous studies have mainly focused on use and found mixed results [8,9]. One finding suggests that the young benefit more from internet use than older people [5], but another study had opposite findings [10]. Skills and access were not adjusted for in these studies, however, and our results suggest that they are of importance. For example, in Italy researchers found that a substantial proportion of those aged 50 years or older were not digitally skilled [28]. Because skills seem to be crucial for older people to benefit from online services, training should be provided for them [29]. A study found that older people were satisfied with ICT training that included working in couples, practicing with the device, choosing what to learn, and practicing material that facilitates communication and learning [30].

Differences in education level could be partly explained by differential access, skills, and use. Previously, lower education has been found to be associated with fewer benefits from internet use [5,10] and lower use of governmental e-services [8]. Computer nonusers have been found to have less education, higher unemployment, lower annual income, and poorer health and be less likely to have memberships in community organizations or perform volunteer work than their counterparts [9].

Previous studies have found mixed results regarding findings related to urbanization level. Rural participants are less likely to have regular access to the internet or manage personal health information online and email health care providers in the United States [31]. However, it has been found that rural patients engage digital rehabilitation means more than urban patients [32]. In Sweden, primary care choice reform in 2010 seemed to have negative effects on geographic equity; the effects do not appear to be very large but they concern some indicators of socioeconomically less advantaged areas [33]. The introduction of electronic online services may face special challenges in rural areas [34]. It has been suggested that rural online service implementation only leads to sustainable adoption (ie, it sticks) when the implementation carefully considers and aligns the content (the clicks), the preexisting structures in the context (the bricks), and the interventions in the implementation process (the tricks) [35].

Our findings that gender was unrelated to experienced benefits are supported by an earlier study from the Netherlands, where there were no gender differences related to who benefits from internet use [5]. Correspondingly, in Finland there were no gender differences in the use of governmental online services [8].

Deursen and Helsper [5] suggested that economic benefits of digital services are related to economic resources such as education, whereas social benefits are more related to social resources. Our findings give only partial support to this, given that satisfaction with relationships and keeping in touch with friends and relatives were associated with perceived collaboration benefits but also associated with other forms of benefits. Correspondingly, education was associated with economic benefits but also with health benefits and did not remain significant after adjusting for ICT variables. A previous study has found support for this idea showing that differences in economic outcomes were related to economic resources such as education and income, whereas differences in social outcomes were related to social resources such as marital status [5].

### Strengths and Limitations

Previous studies have mainly focused on first-level digital divide or exclusion showing that demographics, health, socioeconomic position, and social participation are associated with the use of internet and online services. However, it has been suggested that shifting research focus from the first-level divide to the third-level divide and examining the benefits from online services and the inequalities in these outcomes is important [16,17]. Research related to social determinants of benefits is especially lacking [17]. Focusing on aspects of the third-level divide or exclusion and factors related to them is a strength of this study.

This study used a large random sample representative of the Finnish population. However, it relied on self-reported measures, which may lead to problems associated with an inflation of the strengths of associations and with common method variance. To minimize problems with self-reports, we used measures that showed good reliability. Our study was cross-sectional, thus we cannot draw any conclusions about causality. Moreover, although we controlled for many factors, we cannot rule out the possibility of residual confounding. In addition, there are many possible determinants affecting the use and benefits of online services that we did not examine. For example, we did not examine attitudes toward ICT and motivation to use it, satisfaction with care provider, or personality. Future studies should examine these factors and the different types of health internet users (ie, a recent study found 6 different types: learners, pragmatists, skeptics, worriers, delegators, and adigitals [36]).

Finland is among the forerunners in the digitalization of health care and social welfare services [33]: the national digital Kanta services are unique in the world, and tax-financed universal health care is provided for all residents. Therefore, generalizing our findings to countries with other types of health care systems or online services should be done with caution.

In our sample the respondents were older, had more education, and were more often women than the eligible population [18]. One reason for this was that we used double picking probability for those 75 years of age or older to guarantee a sufficient group size for older people, given that they are an important group suggested to be at risk of digital divide and exclusion. To tackle possibly biased results, we used inverse probability weighting based on age, gender, marital status, education level, living region, and degree of urbanization of the residential municipality.

### Conclusions

Our findings suggest that access to electronic online services, skills to use them, and the extent of use play crucial roles in perceiving benefits from these services. Skills seem to be the most central element. Moreover, health, financial hardship, and participation-related variables seem to be important as well. Thus, it seems that there is a significant risk of digital exclusion among economically disadvantaged people, those with poor health, and those who are socially isolated. In times when services are increasingly provided by electronic means, this digital divide may predispose people who do not perceive benefit from online services to be excluded from those services.

To ensure that the population, particularly those who are older, in poor health, socially isolated, or of low socioeconomic position, can equally benefit from online health services, access, skills, and possibilities to use these services must be provided. These groups are at the highest risk of digital exclusion and have the highest need for health and social welfare services. Promoting use would also increase benefits for organizations by enabling more users for online services.

Providing services online has the potential to widen social and health inequalities among the population. Widespread expansion calls for rigorous consideration of interventions aimed at tackling the negative effects that can arise from providing health and social care services online and promoting equal opportunities and capabilities among the population [37]. In accordance with Öberg et al [38], targeting training toward vulnerable groups such as senior citizens and people with poor health, lower levels of education, or social isolation may help to ensure that online health services are accessible and can reach a wide population and improve client involvement in their own care. Moreover, organizations should consider offering instruction and support services to improve patient engagement [29].

## Appendix

Multimedia Appendix 1 Questionnaire items.

## Abbreviations

ICT: information and communication technologies
EM: estimated mean
