# Supplementary material for: Digital Divide in Perceived Benefits of Online Health Care and Social Welfare Services: National Cross-Sectional Survey Study
Source: J Med Internet Res. 2020 Jul 7;22(7):e17616. doi: 10.2196/17616 (PMC7381057; doi:10.2196/17616)
Supplement: Multimedia Appendix 1 [file jmir_v22i7e17616_app1.docx]

**Multimedia appendix 1.**

The items of the measurement scales

*Dependent variables*

**Health benefits:**

*What do you perceive as the most essential benefits of online social and health care services? Online services…*

1. help people self-manage their health / well-being
2. help people assess the risks / need for services related to health / well-being
3. support finding and choosing services most suitable for the person
4. help people promote the health / well-being of their next of kin/ dependant
5. help the maintenance of the security of customer and patient data
6. help the adaptation of services to different customer groups

Response options:

1. Completely disagree
2. Somewhat disagree
3. Don’t agree or disagree
4. Somewhat agree
5. Completely agree

**Economic benefits:**

*What do you perceive as the most essential benefits of online social and health care services? Online services…*

1. provide useful reminders (e.g. the time of reception, laboratory tests, renewing prescriptions, etc.)
2. facilitate getting appropriate services regardless of time and place
3. save time or money that making transactions In person would take (travel costs, working hours)
4. enable shorter and more efficient care (e.g. avoiding overlap of examinations and data collection, collecting preliminary data electronically already before the appointment)

Response options:

1. Completely disagree
2. Somewhat disagree
3. Don’t agree or disagree
4. Somewhat agree
5. Completely agree

**Collaboration benefits:**

*What do you perceive as the most essential benefits of online social and health care services? Online services…*

1. facilitate people’s participation in the treatment of their health / well-being issue in cooperation with professionals
2. support collaboration and information flow between the patient / customer and the social and health personnel in charge of the person’s treatment
3. help patients / customers in following where their health and customer data have been processed

Response options:

1. Completely disagree
2. Somewhat disagree
3. Don’t agree or disagree
4. Somewhat agree
5. Completely agree

*Independent variables:*

**Access to online services**

*What factors make it impossible/difficult for you to use the online social and health care services*

1. I do not have a computer and internet connection of my own
2. Data connections are poor in my area
3. The services I need are not available electronically
4. It is hard to find electronic services
5. The electronic services are not accessible to me e.g. due to my visual impairment
6. Electronic services are not available in my native language
7. I cannot carry out transactions electronically on behalf of a third party, although it would be necessary

Response options:

1. Completely agree
2. Somewhat agree
3. Don’t agree or disagree
4. Somewhat disagree
5. Completely disagree

**Skills to use online services**

*What factors make it impossible/difficult for you to use the online social and health care services*

1. I do not have sufficient technical skills to use the e-services
2. Electronic services are difficult to use
3. I have not received support for using the online services of social and health care
4. I cannot get help anywhere in the event of a technical problem
5. The terms of use are vague and too long ("I have read and agree to these terms")

Response options:

1. Completely agree
2. Somewhat agree
3. Don’t agree or disagree
4. Somewhat disagree
5. Completely disagree

**The extent of use of online services**

*How have you been doing the following things during the past year?* You can choose between the traditional (paper, visit or phone call) and the electronic service use (via mobile devices or computer).

1. Followed / measured my personal well-being and health, and its changes regularly (e.g. food diary, weight, blood pressure, welfare or activity bracelet)
2. Searched for reliable information to promote my personal health and well-being or sought information about diseases, their symptoms and treatment
3. Taken risk tests, symptom assessments, a health check or a written evaluation of my personal functional capacity
4. Asked health- or welfare-related advice from a professional
5. Sought information on health or social services in my region
6. Compared the price, queues for, or the quality of the services offered
7. Made an application for social service or social assistance
8. Scheduled an appointment in a social or health care service
9. Requested a renewal of a medical prescription
10. Received a decision on a social service or social assistance
11. Viewed the patient/customer data recorded by professionals about me
12. Made requests to correct errors I have personally noticed in my patient / customer data
13. Received laboratory or other test results
14. Disclosed my personal measurements or other information to professionals related to the customer relationship
15. Received advice or guidance from health care or social welfare professionals, e.g., based on the test results or the monitoring data I provided them
16. Had an appointment with a health or social care professional

Response options:

1. No
2. Yes traditionally
3. Yes electronically
